# PCLLA-nanoHA Bone Substitute Promotes M2 Macrophage Polarization and Improves Alveolar Bone Repair in Diabetic Environments

**DOI:** 10.3390/jfb14110536

**Published:** 2023-10-30

**Authors:** Dandan Wang, Ling Wei, Jialin Hao, Weifeng Tang, Yuan Zhou, Chenguang Zhang, Jinming Wang

**Affiliations:** 1 Department of Pediatric Dentistry, Peking University School and Hospital of Stomatology, Beijing 100081, China; wangdandankq@pkuss.bjmu.edu.cn; 2 Third Clinical Division, Peking University School and Hospital of Stomatology, Beijing 100081, China; v0mysun@163.com (L.W.); tangweifeng@126.com (W.T.); 3 Department of Prosthodontics, Stomatological Hospital and Dental School of Tongji University, Shanghai Engineering Research Center of Tooth Restoration and Regeneration, Shanghai 200072, China; jialinhao8883@126.com; 4 Shenzhen Stomatological Hospital, Southern Medical University, 1092 Jianshe Road, Luohu District, Shenzhen 518001, China; 5 Guangdong Provincial Key Laboratory of Stomatology, Department of Oral Implantology, Guanghua School of Stomatology, Hospital of Stomatology, Sun Yat-sen University, Guangzhou 510275, China

**Keywords:** bone immunity, diabetes, hydroxyapatite, macrophage differentiation, poly (caprolactone-co-lactide)

## Abstract

The utilization of bioresorbable synthetic bone substitutes with immunomodulatory properties has gained significant attention in dental clinical applications for the absorption of alveolar bone induced by orthodontic treatment. In this study, we developed two distinct materials: a conventional hydroxyapatite (HA) bone powder comprised of hydroxyapatite particles and nanoHA embedded within a poly(caprolactone-co-lactide) (PCLLA) elastomeric matrix. We assessed the physicochemical characteristics of the bone substitute, specifically focusing on its composition and the controlled release of ions. Our findings show that PCLLA-nanoHA has deformable properties under 40 N, and a significant release of Ca and P elements was noted after 7 days in aqueous settings. Moreover, at the protein and gene expression levels, PCLLA-nanoHA enhances the capacity of macrophages to polarize towards an M2 phenotype in vitro. In vivo, PCLLA-nanoHA exhibits comparable effects to standard HA bone powder in terms of promoting alveolar bone regeneration. Extensive investigations reveal that PCLLA-nanoHA surpasses the commonly employed HA bone powder in stimulating bone tissue repair in diabetic mice. We have identified that PCLLA-nanoHA regulates macrophage M2 polarization by activating the PI3K/AKT and peroxisome proliferator-activated receptor gamma (PPAR) signaling pathways, thereby facilitating a favorable local immune microenvironment conducive to bone repair and regeneration. Our findings suggest that PCLLA-nanoHA presents itself as a promising bioresorbable bone substitute with properties that promote macrophage M2 polarization, particularly in the context of regulating the local microenvironment of alveolar bone in diabetic mice, potentially facilitating bone tissue regeneration.

## 1. Introduction

Orthodontic treatment is a commonly employed method for correcting dental misalignments and improving occlusion that commonly involves tooth extraction, tooth movement, and the application of force. These therapeutic methods aim to alter the position and occlusal relationship of the teeth by exerting forces. However, improper force application or inadequate treatment techniques may lead to localized skeletal damage and the occurrence of complications, including bone dehiscence and fracture. Bone dehiscence, a severe complication, refers to the destruction of the surrounding bone and the formation of a bony window during orthodontic treatment. The presence of such bony windows may result in decreased tooth stability, root exposure, and restricted tooth movement. Timely repair of alveolar bone defects caused by orthodontic treatment is of great significance in prolonging the lifespan of natural teeth and promoting oral function. This is because bone dehiscence, which refers to the exposure of the tooth root due to a lack of bone coverage, can increase the risk of periapical infections and tooth loss. Additionally, bone fractures, another common complication during orthodontic treatment, can result in cracks or fractures in the bone surrounding the tooth roots, potentially leading to tooth instability and restricted movement. Therefore, repairing alveolar bone defects promptly is crucial for maintaining oral health and functionality.

Diabetes mellitus is a chronic metabolic disorder characterized by hyperglycemia that affects various organs and systems, including the oral cavity [1,2]. Dental complications, in particular, are commonly observed in diabetic patients [3,4,5]. Alveolar bone loss caused by bone fenestration and bone dehiscence is a complex biological process that involves both osteoclastic bone resorption and osteoblastic bone formation [6]. However, the use of biomaterials to promote the repair and regeneration of alveolar bone in diabetic patients has been a challenging and extensively researched area.

Recently, there has been growing interest in developing new therapies for diabetes-induced alveolar bone loss using biomaterials that can modulate the immune response and promote tissue regeneration [7]. One promising approach is to use biomaterials that can regulate macrophages activity, as these cells play a critical role in the immune response and tissue repair [8,9]. Macrophages can polarize into different functional states depending on the microenvironment, and their phenotype have a significant impact on tissue homeostasis [10]. Macrophages are a type of white blood cell that play a crucial role in the immune system’s defense against infections and tissue injury [11]. They are phagocytic cells that can engulf and eliminate foreign particles, damaged cells, and microorganisms [12]. In addition to their role in innate immunity, macrophages also play a critical role in tissue homeostasis and repair. They can release various cytokines, chemokines, and growth factors that regulate inflammation, cell proliferation, and differentiation.

Macrophages are heterogeneous cells that can polarize into different functional states depending on the microenvironment and stimuli they encounter. Two main polarization states have been identified: classically activated (M1) and alternatively activated (M2) macrophages. M1 macrophages are pro-inflammatory cells that produce high levels of cytokines, such as interleukin-1β (IL-1β), interleukin-6 (IL-6), and tumor necrosis factor-α (TNF-α). They also produce reactive oxygen species (ROS) and nitric oxide (NO) that can kill pathogens but also cause tissue damage [13,14]. M2 macrophages, on the other hand, are anti-inflammatory cells that produce cytokines, such as interleukin-10 (IL-10) and transforming growth factor-β (TGF-β). They also participate in tissue repair and remodeling by producing extracellular matrix proteins and growth factors [15]. Therefore, the modulation of macrophage activity towards an anti-inflammatory phenotype using biomaterials holds promise as an approach to managing alveolar bone loss, particularly in a diabetic environment [16,17].

In this study, we investigate the therapeutic potential of a novel biomaterial that can regulate macrophage polarization and promote tissue regeneration. NanoHA embedded in a poly(caprolactone-co-lactide) (PCLLA) elastomeric matrix is assessed in this study given its biocompatibility, osteoconductivity, bioactivity, mechanical properties, resorbability, and potential applications in dental clinical settings [18,19,20]. The soft material was created by combining nano-hydroxyapetite and the three-armed rubbery polymer PCLLA. The many surface hydroxyl groups on HA and the end hydroxyl groups at the PCLLA arms create hydrogen bonds in the resulting material, contributing to the creation of a cross-linked network. The combined features of HA and PCLLA confer good biocompatibility, biodegradability, and osteoconductivity on the resulting hybrid material. Most significantly, the hydrophobic PCLLA section gives the material outstanding anti-collapse properties in water conditions, and the distinctive softness provides the user with an easy-to-operate experience. Specifically, we examine the effects of this biomaterial on macrophages polarization and bone remodeling in vitro and in vivo using an ob/ob mouse model.

Our results demonstrate that this biomaterial can modulate macrophage polarization towards an anti-inflammatory phenotype and promote bone regeneration, suggesting its potential as a new therapeutic option for managing alveolar bone loss, including diabetes-induced conditions. Our findings may offer novel insights into the involvement of macrophages in the process of alveolar bone regeneration and could potentially pave the way for the development of innovative therapies for the treatment of bone fenestration and bone dehiscence resulting from orthodontic treatment.

## 2. Materials and Methods

### 2.1. Synthesis of a PCLLA-nanoHA Bone Substitute

First, nanoHA was synthesized using the traditional hydrothermal method according to the literature [21]. Briefly, the (NH_4_)_2_HPO_4_ solution was added dropwise into the Ca(NO_3_)_2_·4H_2_O solution based on ta Ca/P molar ratio of 1.67. Then, the pH value of the solution was kept at about 10 by adding NH_3_·H_2_O, and the solution was stirred for 30 min. Afterward, the mixed solution was transferred into a Teflon-lined stainless-steel autoclave, heated to 140 °C, and maintained for 24 h. The slurry was washed with deionized water four times to remove impurities. Next, the slurry was filtered, freeze-dried for 24 h, and then sifted with a sieve to produce the nanoHA. Second, the three-armed copolymer poly(L-lactide-co-caprolactone) (PCLLA) was synthesized using trimethylolpropane initiating ring-opening polymerization of L-LA and CL monomers according to a molar ratio of 1:1. Under the protection of dry nitrogen flow, L-LA, CL, trimethylolpropane, and the catalyst were subsequently added to a flask equipped with a stirring bar. After several pump-thaw cycles, the flask was sealed under vacuum and then immersed in an oil bath at 130 °C. After 48 h, the crude product was dissolved with dichloromethane and precipitated in cold ethanol three times to afford the product PCLLA. Finally, the composite was obtained by mixing HA and rubbery PCLLA with a mass ratio of 1:1 in chloroform. After sonicating for half an hour, the mixture solution was heated to remove the solvent. The obtained product was further dried under vacuum at 70 °C for 24 h [22]. The sterilized product is then used for subsequent in vitro and in vivo experiments after Co60 gamma-ray irradiation.

### 2.2. Scanning Electron Microscopy

The polylactic acid bone cement samples were immersed in a modified simulated body fluid and incubated at 37 °C in an incubator for 0, 7, and 21 days, followed by natural air drying. To enhance the conductivity of the samples, ion sputtering was performed using an ion sputter coater. The surface morphology of the materials was observed and captured at the desired magnification using a field emission scanning electron microscope (SEM, ThermoFisher, Waltham, MA, USA) equipped with an X-ray energy-dispersive spectroscopy (EDS) analyzer to determine the elemental composition.

### 2.3. X-ray Photoelectron Spectroscopy

The polylactic acid bone cement samples were immersed in a modified simulated body fluid and incubated at 37 °C in an incubator for 0 and 14 days. After complete drying, the samples were cut into blocks with dimensions of 5 mm × 5 mm × 1 mm. The samples were securely attached to sample holders using regular double-sided adhesive tape. X-ray photoelectron spectroscopy (XPS, ThermoFisher, Waltham, MA, USA) was employed to analyze the chemical composition of the material surface. Care was taken during the entire process to ensure the cleanliness of the sample surface.

### 2.4. Mechanical Property

The polylactic acid bone cement samples were prepared as standard specimens, immersed in a modified simulated body fluid, and incubated at 37 °C in an incubator for 7 days. Subsequently, the tensile modulus of the samples was assessed utilizing the electronic universal tensile tester (WD-T-10, JOTECH, Shanghai, China), yielding force-deformation curves and ultimately leading to the computation of the tensile modulus. The polylactic acid bone cement samples were prepared as equilateral triangles and squares with 3 cm sides and circles with 3 cm to assess the material ductility.

### 2.5. Cell Culture

Femurs and tibias of healthy C57 mice or ob/ob mice (Beijing Vital River Laboratory, Beijing, China) aged 6–8 weeks were separated, while their epiphysis sides were removed. Bone marrow cavity was rinsed with DMEM medium (Cyangen Bioscience, Beijing, China) for 3–4 times until the bone turned white. The solution obtained after rinsing was centrifuged and then resuspended with medium containing granulocyte-macrophage colony-stimulating factor (GM-CSF, Peprotech, Nanjing, China) at a concentration of 20 ng/mL. Cells were cultured for 7 days, and the medium containing GM-CSF was changed every 2 days. Bone marrow-derived macrophages (BMDM cells) were seeded on 48-well plates at a density of 2 × 10^4^ to evaluate cell proliferation. The experimental group was cultured in the material extraction solution, while the control group was cultured in DMEM with 10% Fetal Bovine Serum (FBS) and 1% Penicillin-Streptomycin Solution (P/S). HA/PCLLA was introduced in culture medium for 2 days and used to prepare a material extraction solution environment by gradual dissolution and nanoparticle release. The negative control group was cultured in a medium containing 0.064% phenol. Cell proliferation was assessed on days 1, 3, 5, and 7 using a cell counting kit-8 assay (Dojindo, Tabaru, Japan) with optical density measured at 450 nm.

### 2.6. Immunofluorescence Analysis

Macrophage polarization was assessed using immunofluorescence staining. Cells were seeded on confocal dishes and cultured either with extraction medium or DMEM with 10% FBS. The samples were incubated overnight at 4 °C with primary antibody, including anti-mannose receptor (CD206) (abcam), which was dissolved in 5 wt% BSA in PBS. After rinsing to remove excess antibody, the cells were incubated with the corresponding secondary antibody for 1 h. Cytochemical staining of the cytoskeleton was performed using Phalloidin (Solarbio, Beijing, China), while nuclei were stained using 4′,6-diamidino-2-phenylindole (DAPI; Sigma, Darmstadt, Germany). Confocal laser scanning microscopy (Leica, Wetzlar, Germany) was used to capture images.

### 2.7. RT-qPCR

In 6-well culture dishes, 5.0 × 10^5^ BMDM cells were plated and cultured for 3 days. The cells were treated with an extraction solution containing HA and PCLLA. Control cells were grown in a medium containing 0.064% phenol. The total RNA from the two groups of cells was then isolated and reverse transcribed into cDNA using RNA extract and the PrimeScript RT reagent kit (Takara Co., Shiga, Japan). The cDNA was then amplified using a real-time quantitative polymerase chain reaction (RT-qPCR) under the following amplification conditions: 95 °C for 30 s, followed by 39 cycles of 95 °C for 5 s and 60 °C for 30 s. Optical 96-well reaction plates and optical adhesive films (Thermo Fisher Scientific, Waltham, MA, USA) were used for PCR. Each well received 20 L of the PCR mixture, which included 8 μL of FastStart Universal SYBR Green Master Mix (Rox), 10 L of RNase-free water, 1 μL of template cDNA, and 1 μL of primer. The following cycling parameters were used for PCR amplification: 15 min at 95 °C (heat activation step), then 40 cycles of 15 s at 95 °C and 1 h at 60 °C. Thermo Fisher Scientific’s QuantStudio Design & Analysis Desktop Software (version 1.5.2) was used to examine the data. The levels of mrc1, arg-1, and iNOS gene expression were estimated using cycle threshold (Ct) values relative to the endogenous housekeeping control gene (GAPDH). The differences in gene expression levels between groups were statistically examined. The primer sequences are presented in Table 1. Glyceraldehyde-3-phosphate dehydrogenase (GAPDH) served as the internal control.

### 2.8. In Vivo Experiment

All animal experiments were conducted in strict compliance with the Animal Ethics Committee of Peking University, following the approved guidelines (IACUC number LA201108). The following procedures were performed on C57 mice and ob/ob mice (Beijing Vital River Laboratory, Beijing, China). A circular hole measuring 1.2 mm in diameter was carefully drilled into the mandible bone. The PCLLA-nanoHA bone substitute underwent a softening process by heating it with a microwave emitter at 50 °C for 5 min prior to filling. This heating step made the material ductile and easily moldable to match the shape of the bone defect. Subsequently, HA bone cement material was implanted into the hole, and the area was tightly sutured. After a period of 2 weeks and 4 weeks, the mice were sacrificed in order to obtain their femurs for further assessments.

### 2.9. Microcomputed Tomography Scan

The specimens were subjected to analysis using a micro-computed scanner, specifically the Bruker Skyscan 1276 from Germany. The scanning process utilized a resolution protocol of 21.2 μm (voltage: 100 kV; current: 200 μA; Cu filter; integration time: 1500 ms). The microCT images were reconstructed using CTvox software (version 3.0.0) (Bruker Daltonics, Bremen, Germany). To quantify the regenerated bone, the volumes of regenerated bone tissues within the bone defect area were measured. The total volume was defined as the original bone defect with dimensions of 1.2 mm. From these measurements, the bone volume/total volume ratio (BV/TV) and trabecular number (Tb·N) were calculated.

### 2.10. RNA Sequencing

Total RNA was isolated using TRIzol reagent (Invitrogen, Carlsbad, CA, USA) as per the manufacturer’s instructions. Quantification of RNA was performed using NanoDrop ND-1000 (NanoDrop, Wilmington, DE, USA), and RNA integrity was assessed using Bioanalyzer 2100 (Agilent, Santa Clara, CA, USA). Poly(A) RNA was purified from 1 μg total RNA using Dynabeads Oligo(dT)25-61005 (Thermo Fisher, Waltham, CA, USA) through two rounds of purification. The fragmented RNA was reverse-transcribed into cDNA using SuperScript™ II Reverse Transcriptase (Invitrogen, cat. 1896649, Waltham, MA, USA). U-labeled second-stranded DNAs were synthesized from the cDNA using *E. coli* DNA polymerase I (NEB, cat.m0209, Thermo Fisher, Waltham, MA, USA), RNase H (NEB, cat.m0297, USA), and dUTP Solution (Thermo Fisher, cat.R0133, USA). Blunt ends were generated by adding an A-base to each strand to facilitate ligation to indexed adapters. Fragments were ligated to single- or dual-index adapters with T-base overhangs, followed by size selection using AMPureXP beads. The ligated products were then amplified by PCR after treatment with heat-labile UDG enzyme (NEB, cat.m0280, USA) to remove U-labeled second-stranded DNAs. The resulting cDNA library had an average insert size of 300 ± 50 bp. Paired-end sequencing (PE150) of the cDNA library was performed on an Illumina Novaseq™ 6000 platform (LC-Bio Technology CO., Ltd., Hangzhou, China) following the vendor’s protocol.

### 2.11. Bioinformatics Analysis of RNA-seq

Fastp software (version 0.23.1) (HaploX, Shenzhen, China) was used to remove adaptor contamination, low-quality bases, and undetermined bases from the reads. Sequence quality was verified using fastp. HISAT2 was employed to map the reads to the reference genome of *Homo sapiens* GRCh38. The mapped reads were assembled using StringTie, and a comprehensive transcriptome was reconstructed by merging transcriptomes from all samples using gffcompare. Expression levels of all transcripts were estimated using StringTie. FPKM values were calculated to quantify mRNA expression levels. Differentially expressed mRNAs were identified based on fold change and statistical significance using the edgeR package in R.

### 2.12. Statistical Analysis

Data are expressed as means ± standard deviation (SD) and were analyzed using SPSS software (version 15.0; SPSS Inc., Chicago, IL, USA). The level of significance was determined via one-way ANOVA followed by the Student–Newman–Keuls post hoc test for multiple comparisons. Values of *p* < 0.05 were considered statistically significant.

## 3. Results

### 3.1. Characterization of PCLLA-nanoHA Bone Substitute

**Figure 1:**
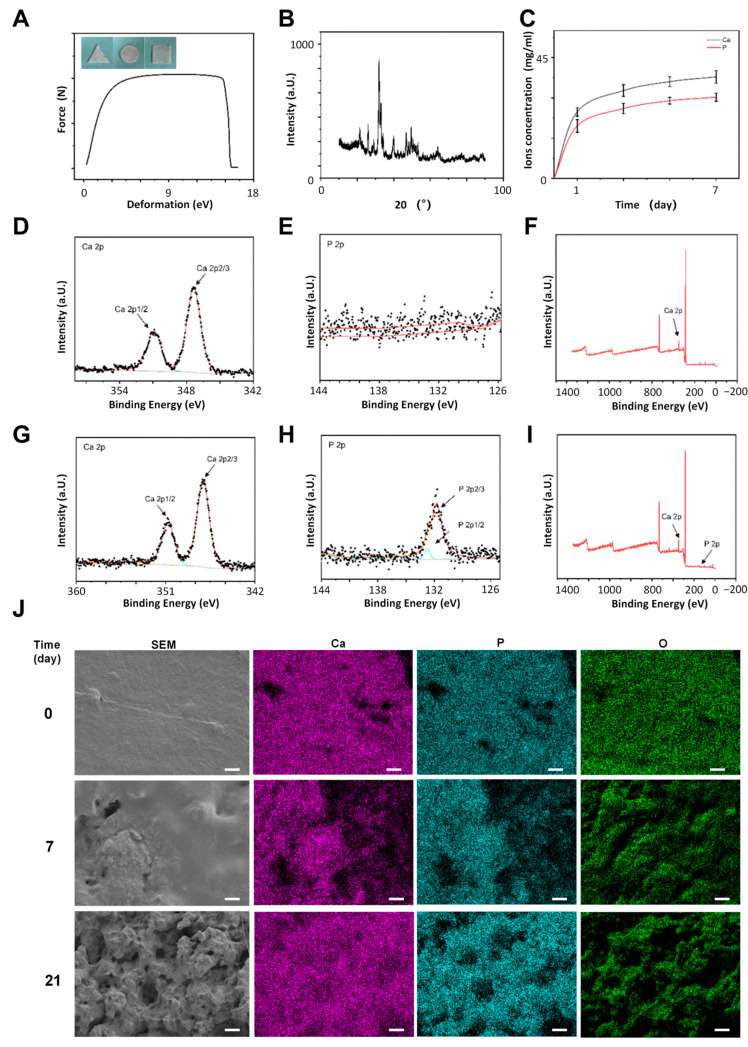
Characterization of the components in HA/PCLLA bone substitute. (**A**) The tensile curve represents the ductility of the composite material. (**B**) XRD spectra of HA/PCLLA. (**C**) The concentrations of Ca and P in SBF solution. (**D**–**I**) XPS analysis showing the significant peaks associated with the gradual exposure of Ca and P ions upon immersion. (**J**) SEM characterization and compositional analysis revealing the gradual degradation and exposure of HA components in different time-immersed SEM images. Scale bar = 10 μm.

In order to investigate the mechanical properties of the PCLLA-nanoHA material, a tensile test was performed. As depicted in Figure 1A, the force-deformation curve for the material was recorded, leading to the calculation of a tensile modulus value of 28.5 MPa. This observation suggests that the incorporation of PCLLA enhances the material’s ductility, rendering it amenable to shaping into diverse configurations. Such an improvement in ductility enhances the clinical maneuverability of the material. Furthermore, X-ray diffraction (XRD) analysis revealed a prominent characteristic peak of hydroxyapatite at 31.8° for the PCLLA-nanoHA composite (Figure 1B). Moreover, ion release experiments demonstrated that after a 7-day immersion period, PCLLA-nanoHA exhibited a certain degree of capability for releasing calcium and phosphate ions (Figure 1C). The degradation behavior of PCLLA-nanoHA was investigated using scanning electron microscopy (SEM) and X-ray photoelectron spectroscopy (XPS). XPS analysis confirmed the presence of Ca element peaks in both 0-day and 14-day immersion groups, while the P element peak was only observed in the 14-day immersion group, suggesting the exposure of HA due to PLLA degradation (Figure 1D–I). SEM images revealed different materials at various immersion times, indicating the gradual degradation of PCLLA and the subsequent exposure of HA components (Figure 1J). The material surface appeared smooth and homogeneous at 0 days of immersion. However, with increased immersion time, PLLA will degrade into free lactic acid, which is metabolized by the body, exposing the components of nanoHA in it. EDX analysis showed an increase in the molar percentage of P element on the material surface from 2.43% (0 days) to 4.77% (21 days). These combined results provide valuable insights into the degradation behavior of the PCLLA-nanoHA bone substitute, forming a foundation for further investigations and applications in bone tissue engineering.

### 3.2. Macrophage M2 Polarization and Alveolar Bone Repair

**Figure 2:**
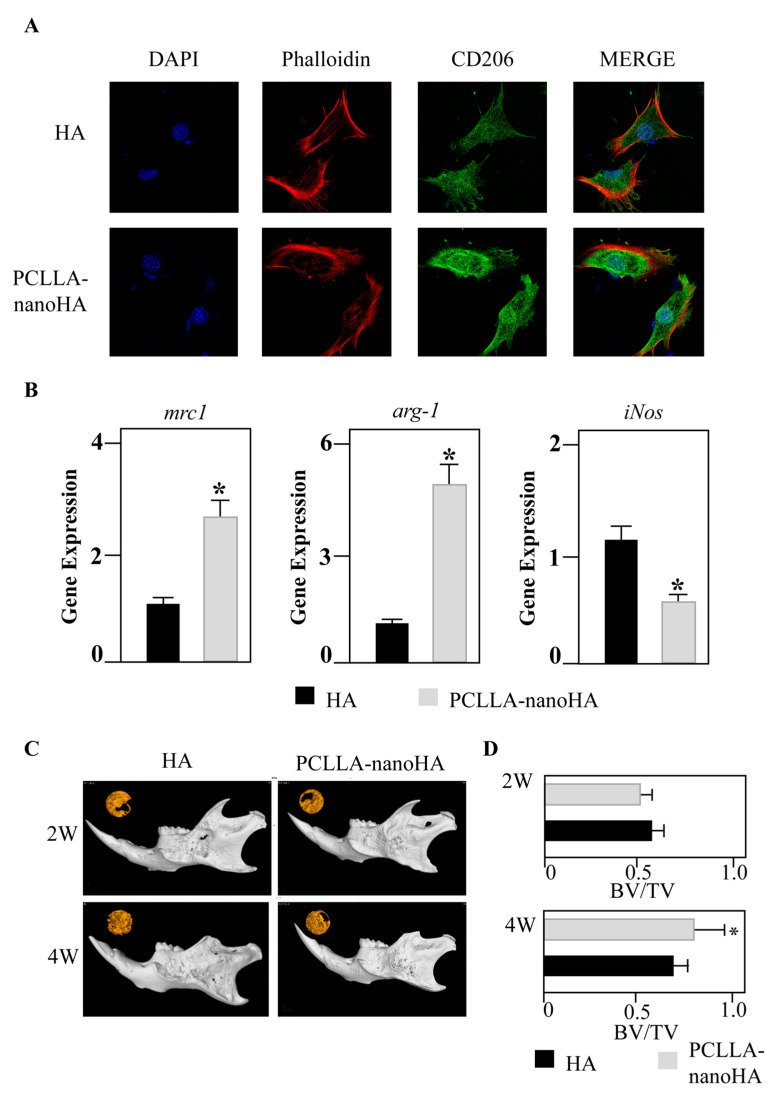
The PCLLA-nanoHA material promotes macrophage M2 polarization and alveolar bone repair. (**A**) Immunofluorescence staining demonstrating significantly higher expression of CD206 in the PCLLA-nanoHA group compared to the HA group. (**B**) Macrophages cultured on material surfaces for 3 days show upregulation of M2-related genes mrc1 and arg-1 in the PCLLA-nanoHA group, while the expression of the inflammatory macrophage gene iNOS is significantly downregulated. (**C**,**D**) MicroCT scans of 1.2 mm mouse alveolar bone defect models implanted with HA or PCLLA-nanoHA material at 2 weeks and 4 weeks, demonstrating favorable tissue repair outcomes for both materials. * *p* < 0.05; two-tailed unpaired Student’s *t*-test.

Building upon the characterization results, the material was examined for its effect on macrophage polarization and alveolar bone repair. A cell counting kit-8 (CCK-8) assay (Figure S1) showed both HA and PCLLA-nanoHA material had similar biocompatibility to cultured macrophages. Immunofluorescence staining revealed a significant increase in the expression of CD206, a marker associated with M2 polarization, in the PCLLA-nanoHA group compared to the HA particle group (Figure 2A).

To further explore the underlying mechanisms, macrophages cultured with HA/PCLLA were subject to gene expression analysis. After three days of culture, the PCLLA-nanoHA group exhibited upregulated expression of M2-related genes, including mannose receptor (mrc1) and type I arginase (arg-1), while the expression of the inflammatory macrophage gene nitric oxide synthase (iNOS) was downregulated (Figure 2B). These findings highlighted the capacity of the PCLLA-nanoHA material to promote M2 polarization in macrophages, contributing to enhanced tissue repair.

Moreover, in a mouse alveolar bone defect model, both the HA and PCLLA-HA materials demonstrated excellent outcomes in terms of tissue repair. MicroCT scans conducted at 2 weeks and 4 weeks post-implantation showed favorable repair effects for both materials, indicating their potential as bone substitutes in alveolar bone regeneration (Figure 2C,D). Multiple studies have shown that macrophages had a close relationship with bone formation.

### 3.3. Comparative Analysis of Macrophage Polarization Characteristics

**Figure 3:**
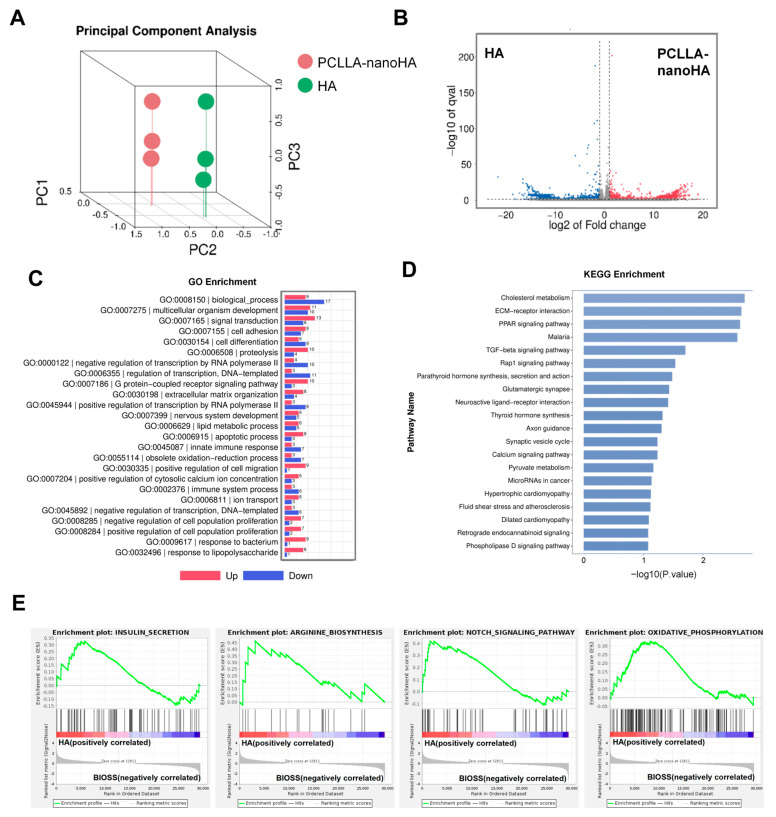
Comparative analysis of macrophage polarization characteristics regulated by PCLLA-nanoHA material and HA. (**A**) PCA analysis indicating differences in macrophage transcriptome characteristics regulated by PCLLA-nanoHA compared to traditional HA powder. (**B**) Volcano plot displaying the differentially expressed genes in macrophages regulated by PCLLA-nanoHA material compared to HA. (**C**) The GO analysis revealed significant enrichment of distinct biological process terms associated with the upregulated genes regulated by PCLLA-nanoHA and HA materials. (**D**) KEEG analysis suggesting significant enrichment of anti-inflammatory and pro-repair signaling pathways in genes upregulated by HA. (**E**) GSEA analysis showing significant regulation of insulin secretion, arginine synthesis, notch, and oxidative phosphorylation signaling pathways by PCLLA-nanoHA.

To gain further insights into the differential effects of the PCLLA-nanoHA material and HA on macrophage polarization, RNA sequencing analysis was performed [23]. This analysis revealed distinct transcriptome characteristics regulated by the PCLLA-nanoHA material compared to the traditional HA powder. Principal component analysis (PCA) demonstrated significant differences in the transcriptome profiles, indicating divergent modulation of macrophage polarization by the two materials (Figure 3A). Volcano analysis displayed the differentially expressed genes in macrophages regulated by the HA material compared to PCLLA-nanoHA, providing valuable information about the gene expression patterns influenced by the PCLLA-nanoHA material (Figure 3B). Functional analysis, including Gene Ontology (GO) analysis and Kyoto Encyclopedia of Genes and Genomes (KEGG) pathway analysis, was conducted to explore the biological processes and signaling pathways affected by the PCLLA-nanoHA material (Figure 3C,D). The classical M2 polarization-associated signaling pathways, including PPARγ and TGFβ, were significantly enriched in the PCLLA-nanoHA group. Additionally, GSEA analyses demonstrated that the PCLLA-nanoHA material upregulated genes associated with anti-inflammatory and pro-repair signaling pathways, such as insulin secretion, arginine synthesis, notch, and oxidative phosphorylation (Figure 3E). These findings provide insights into the molecular mechanisms underlying the observed M2 polarization and tissue repair effects induced by the PCLLA-nanoHA material. Specifically, our study revealed that the PCLLA-nanoHA material promotes M2 polarization of macrophages compared to HA, potentially due to the enrichment of multiple signaling pathway molecules associated with suppressing inflammation and promoting repair [24].

### 3.4. Enhanced Alveolar Bone Repair in Diabetic Mouse Models

**Figure 4:**
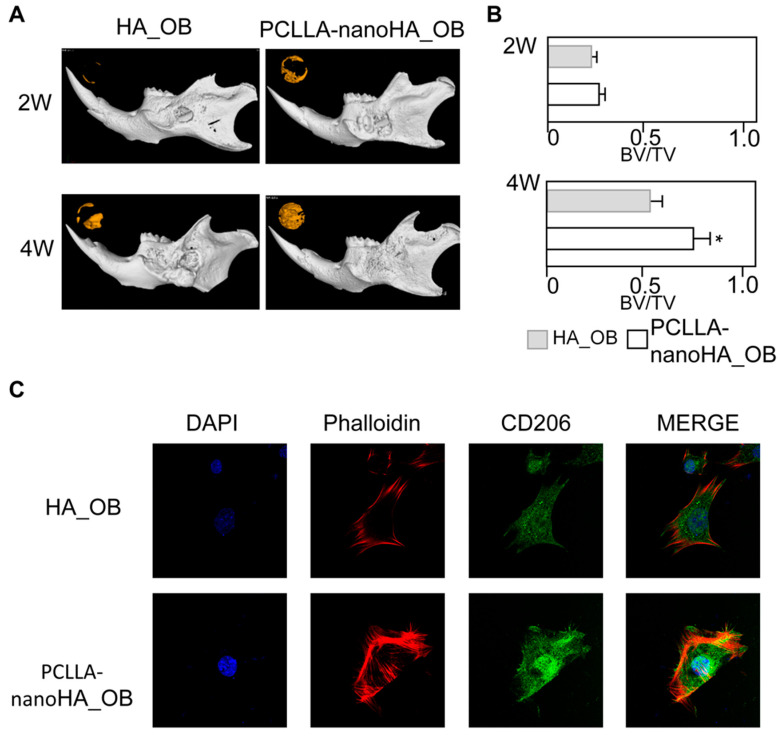
Enhanced alveolar bone repair in diabetic mouse models through promotion of M2 macrophage polarization by PCLLA-nanoHA material. (**A**,**B**) MicroCT scans of alveolar bone defect models in diabetic mice implanted with PCLLA-nanoHA or HA material at 2 weeks and 4 weeks, demonstrating significantly better repair outcomes with HA compared to the PCLLA-HA group. (**C**) Immunofluorescence staining showing significantly higher CD206 expression in macrophages derived from diabetic mice from the PCLLA-nanoHA group compared to the PCLLA-HA group. * *p* < 0.05; two-tailed unpaired Student’s *t*-test.

Given the increased prevalence of diabetes and its impact on impaired bone healing, it was important to investigate the effect of PCLLA-nanoHA material on alveolar bone repair in diabetic mouse models. Ob/ob mice [25] were utilized to establish the alveolar bone defect model, and HA or PCLLA-nanoHA bone graft materials were implanted in the respective groups. MicroCT scans conducted at 2 weeks and 4 weeks post-implantation demonstrated significantly enhanced alveolar bone repair in the PCLLA-nanoHA group compared to the HA group (Figure 4A,B). These findings indicated that the PCLLA-nanoHA material exhibited improved regenerative capacity, particularly in the challenging context of diabetes. Immunofluorescence staining of macrophages derived from diabetic mice further supported these results, showing significantly higher expression of CD206 on the PCLLA-nanoHA material compared to the HA group (Figure 4C). This finding suggests that the PCLLA-nanoHA material was able to effectively promote M2 macrophage polarization even in the diabetic environment, thereby contributing to enhanced alveolar bone repair. Ob/ob mice carry a mutation in the leptin gene, resulting in abnormal expression of leptin. While macrophages are one of the cell types that express leptin, the abnormal expression of leptin in ob/ob mice may impact the function and inflammatory regulation of macrophages, thereby affecting tissue regeneration [26]. However, the specific mechanisms underlying the observed M2 polarization and tissue repair effects induced by the PCLLA-nanoHA material in ob/ob mice require further investigation.

### 3.5. Mechanisms of PCLLA-nanoHA Material in Promoting M2 Polarization of Macrophages from Diabetic Mouse Models

**Figure 5:**
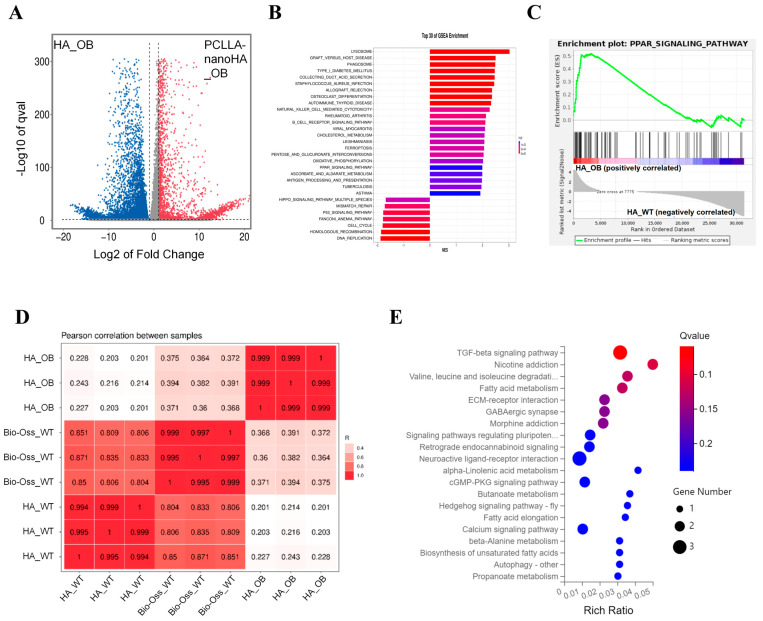
Mechanisms of PCLLA-nanoHA material in promoting M2 polarization of macrophages from diabetic mouse models via multiple signaling pathways. (**A**) Volcano plot displaying the abundance of differentially expressed genes regulated by PCLLA-nanoHA material in macrophages obtained from wild-type (WT) and diabetic mice. (**B**) Enrichment bar plot from GSEA analysis. (**C**) PPAR signaling pathway is upregulated by PCLLA-nanoHA. (**D**) Significant differences in component expression between macrophages obtained from WT and OB mice cultured on PCLLA-nanoHA compared to HA. (**E**) Enrichment results of overlapping KEGG signaling pathway genes in macrophages obtained from WT and OB mice after PCLLA-nanoHA culture.

To unravel the underlying mechanisms of how the nanoHA material can further promote M2 polarization of macrophages derived from ob/ob mice, comprehensive analyses were conducted to investigate the changes in gene expression and signaling pathways influenced by the PCLLA-nanoHA material. The volcano plot demonstrated a significant number of differentially expressed genes regulated by the PCLLA-nanoHA material in macrophages from both wild-type and diabetic mouse models (Figure 5). This highlighted the substantial transcriptomic alterations induced by the PCLLA-nanoHA material in the presence of diabetes.

Further enrichment analysis, including Gene Set Enrichment Analysis (GSEA) and pathway analysis, provided insights into the specific signaling pathways modulated by the PCLLA-nanoHA material (Figure 5B,C). The PCLLA-nanoHA material exhibited a significant upregulation of the PPAR signaling pathway, suggesting its involvement in macrophage polarization and tissue repair. Moreover, the analysis revealed overlapping gene expression changes and enriched signaling pathways in macrophages obtained from wild-type and diabetic mice after exposure to the PCLLA-nanoHA material.

Additionally, Pearson correlation analysis between samples highlighted significant variations in the expression of specific components between macrophages derived from diabetic mice cultured on PCLLA-nanoHA material, which were notably different from those cultured on HA material (Figure 5D). This indicates that the response of macrophages from diabetic mice to the PCLLA-nanoHA material differs substantially from HA material in terms of gene expression or protein abundance. This suggests that the physical characteristics of the substrate have a noticeable impact on the cellular response and behavior of macrophages, which could potentially influence their functional properties and signaling pathways.

Enrichment analysis of the overlapping genes from KEGG signaling pathways in macrophages obtained from WT and OB mice after HA culture further supports the notion that the upregulated genes in OB macrophages on PCLLA-nanoHA material are significantly enriched in signaling pathways associated with promoting reparative macrophage polarization, such as the TGF-β pathway (Figure 5E). The enrichment analysis provides additional evidence that the PCLLA-nanoHA material influences the gene expression profile and signaling pathways in OB macrophages, particularly highlighting the significant enrichment of genes in the TGF-β pathway [27]. This information reinforces the potential role of PCLLA-nanoHA in promoting macrophage polarization towards a reparative phenotype and supports its potential application in tissue repair and regeneration [22].

## 4. Discussion

Classic hydroxyapatite materials have been widely employed in the field of dentistry. In clinical practice, hydroxyapatite bone powder is frequently utilized as a bone regeneration scaffold for jaw bone abnormalities [28]. However, this repair procedure frequently needs a supporting gap, and our enhanced materials have greater flexibility and are ideal for many types of bone defects, particularly during the orthodontic bone opening process [29]. This repair material may handle more oral defect repair conditions. Both conventional HA and nanoHA embedded in a PCLLA matrix are biocompatible materials given that they are compatible with the surrounding tissues and do not elicit adverse reactions or immune responses. This is a critical requirement for any material used in dental clinical settings.

HA, a biomaterial with a composition similar to natural bone mineral, possesses excellent osteoconductive properties [28]. It can provide a scaffold for new bone formation and integration with the surrounding tissues. NanoHA, with its increased surface area and enhanced bioactivity, offers even greater osteoconductive potential. HA and nanoHA have inherent bioactive properties, meaning that they can interact with the surrounding biological environment and stimulate specific cellular responses. This includes promoting osteoblast activity and differentiation, aiding in the formation of new bone, and facilitating osseointegration of dental implants. The addition of the PCLLA elastomeric matrix to HA and nanoHA provides improved flexibility and mechanical properties. This combination offers better handling characteristics, making it suitable for clinical applications, such as dental bone defect repair and augmentation. PCLLA is a resorbable polymer, meaning that it can be gradually broken down and absorbed by the body over time. This property allows for the controlled release of HA or nanoHA, providing sustained bioactivity and facilitating the integration of the implanted material with the surrounding bone [30]. Thus, conventional HA and nanoHA embedded in a PCLLA matrix have a wide range of potential applications in dental clinical settings. They can be used for dental implants, bone grafting procedures, socket preservation after tooth extraction, and restoration of bone defects caused by periodontal disease or trauma.

Our study indicated the ability of the PCLLA-nanoHA material to induce M2 macrophage polarization, which is crucial for tissue repair. Macrophages play a crucial role in inflammation and host defense, and they are classified into two phenotypes, M1 and M2, based on their surface markers and functions [31]. M1 macrophages induce osteoclast generation and enhance their activity through the secretion of proinflammatory cytokines, such as tumor necrosis factor-a (TNF-a), interleukin-6 (IL-6), and interleukin-1B (L-1B), leading to bone resorption. On the other hand, M2 macrophages are involved in tissue repair during the late stage of inflammation. Periodontitis is an infectious disease, and lipopolysaccharide (LPS) on bacterial surfaces can recruit macrophages and polarize them toward the M1 phenotype, producing proinflammatory factors and inducing osteoclast generation and bone loss [32]. Therefore, regulating macrophage polarization to modulate alveolar bone tissue homeostasis is a feasible strategy.

Macrophages promote osteogenic differentiation of bone marrow stromal cells (BMSCs) by secreting bone morphogenetic protein-2 (BMP-2), and polarized M2 macrophages secrete vascular endothelial growth factor (VEGF) to promote angiogenesis [33]. Therefore, in this study, PCLLA-nanoHA is utilized to leverage its immunomodulatory properties in order to selectively induce macrophage phenotypes and promote new bone formation. Previous research has shown that macrophage polarization into different phenotypes is influenced by various cytokines and signaling pathways, and there is interplay between these pathways that result in M1 or M2 polarization [34,35]. Thus, further investigation is needed to elucidate the mechanisms by which PCLLA-HA or -nanoHA materials regulate M2 polarization and induce bone repair.

The PCLLA-nanoHA bone substitute was found to effectively promote M2 polarization of macrophages derived from both wild-type (WT) and diabetic mice compared to conventional HA material primarily through the enrichment of the TGF-β and PPARγ signaling pathways [36,37]. Moreover, in macrophages obtained from diabetic mice, the PCLLA-nanoHA material exhibited an even more significant enrichment in these two signaling pathways, leading to the upregulation of genes associated with these pathways. Consequently, it effectively enhanced alveolar bone repair, particularly in the context of impaired bone healing associated with diabetes.

Impaired bone healing and compromised tissue repair are frequently observed in diabetic conditions. However, our study demonstrated that PCLLA-nanoHA displayed a greater enrichment in the TGF-β and PPARγ signaling pathways in macrophages from diabetic mice. This suggests that nanoHA/PCLLA has a stronger impact on promoting M2 polarization and facilitating bone tissue repair in diabetic individuals [38]. By upregulating genes associated with these pathways, PCLLA-nanoHA enhances the regenerative capacity of macrophages, potentially mitigating the detrimental effects of diabetes on bone healing.

While our study has identified significant alterations in macrophage intracellular signaling, critical inquiries persist regarding the primary receptors engaged in sensing the extracellular matrix microenvironment during the nascent phases of cellular recognition of nanomaterials. The involvement of traditional integrin signaling in the regulation of macrophage polarization remains ambiguous. Regrettably, in this current work, we are devoid of conclusive evidence pertaining to the inception of cellular communication. Our paramount research trajectory, therefore, revolves around elucidating the specific receptors through which macrophages recognize nanoHA.

This enhanced effect of PCLLA-nanoHA may be attributed to its increased degradability, leading to the release and exposure of HA components, such as calcium and phosphate ions [39,40], which are known to activate downstream signaling pathways, including the JAK-STAT, PI3K-Akt pathways, and mitochondrial pathways [41,42]. However, further investigation is needed to elucidate the specific mechanisms by which ions directly regulate macrophages function and how leptin protein responds to ions, ultimately influencing macrophages fate.

## 5. Conclusions

In conclusion, our study has revealed that the PCLLA-nanoHA material effectively promotes M2 polarization of macrophages, thus demonstrating its potential for significantly enhancing alveolar bone repair (Figure S2). Importantly, this finding addresses a critical clinical objective, namely, the treatment of bone fenestration and bone dehiscence commonly associated with orthodontic procedures. Through the meticulous characterization of the material, along with a rigorous comparative analysis of macrophages polarization characteristics and an in-depth investigation into the underlying mechanisms, our research contributes to a comprehensive understanding of the beneficial effects of PCLLA-nanoHA in the context of bone regeneration. This understanding extends to both normal and diabetic conditions, highlighting the material’s versatility and potential relevance for a broad range of clinical applications in the field of orthodontics and beyond.

## Figures and Tables

**Table 1:** Primer sequences utilized for quantitative real-time PCR analysis.

Target Gene	Forward Sequence (5′-3′)	Reward Sequence (5′-3′)
iNOS	GAGCGAGTTGTGGATTGTC	TGAGGGCTTGGCTGAGTGAG
ARG-1	CATATCTGCCAAAGACATC	ACATCAAAGCTCAGGTGAAT
CD206	AGTCAGAACAGACTGCGTG	CAGAGGGATCGCCTGTTCTG
GADPH	CCTCGTCCCGTAGACAATG	TGAGGTCGAAGGGGTCGT

## Data Availability

Not applicable.

## Supplementary Materials

The following supporting information can be downloaded at: https://www.mdpi.com/article/10.3390/jfb14110536/s1, Figure S1: Cell viability and proliferation; Figure S2: Scheme of PCLLA-nanoHA material promotes M2 polarization of macrophages.
