# Supplementary material for: PCLLA-nanoHA Bone Substitute Promotes M2 Macrophage Polarization and Improves Alveolar Bone Repair in Diabetic Environments
Source: J Funct Biomater. 2023 Oct 30;14(11):536. doi: 10.3390/jfb14110536 (PMC10671921; doi:10.3390/jfb14110536)
Supplement: Supplementary file 1 [file jfb-14-00536-s001.zip › jfb-2632395-supplementary.pdf]

Journal of
Functional Biomaterials

MDPI

Supplementary Materials

PCLLA-nanoHA Bone Substitute Promotes M2 Macrophage Polarization and Improves Alveolar Bone Repair in Diabetic Environments

Dandan Wang [1,†], Ling Wei [2,†], Jialin Hao [3,†], Weifeng Tang [2], Yuan Zhou [4,*], Chenguang Zhang [5,*] and Jinming Wang [5,*]

1 Department of Pediatric Dentistry, Peking University School and Hospital of Stomatology, Beijing 100081, China; wangdandankq@pkuss.bjmu.edu.cn
2 Third Clinical Division, Peking University School and Hospital of Stomatology, Beijing 100081, China; v0mysun@163.com (L.W.); tangweifeng@126.com (W.T.)
3 Department of Prosthodontics, Stomatological Hospital and Dental School of Tongji University, Shanghai Engineering Research Center of Tooth Restoration and Regeneration, Shanghai 200072, China; jialinhao8883@126.com
4 Shenzhen Stomatological Hospital, Southern Medical University, 1092 Jianshe Road, Luohu District, Shenzhen 518001, China
5 Guangdong Provincial Key Laboratory of Stomatology, Department of Oral Implantology, Guanghua School of Stomatology, Hospital of Stomatology, Sun Yat-sen University, Guangzhou 510275, China
* Correspondence: zhouyuan2411@gmail.com (Y.Z.); zhangchg9@mail.sysu.edu.cn (C.Z.); wjinm@mail.sysu.edu.cn (J.W.)
† These authors contributed equally to this work.

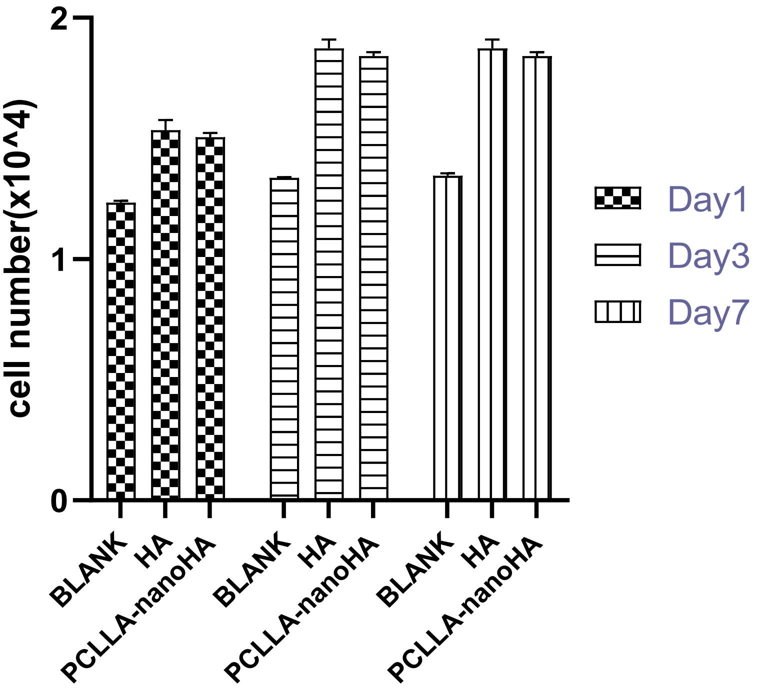

Figure S1. Cell viability and proliferation determined by land CCK-8 assay. No significant differences in cell proliferation were found among the various group by CCK-8 assay after 1, 3 or 7 days.

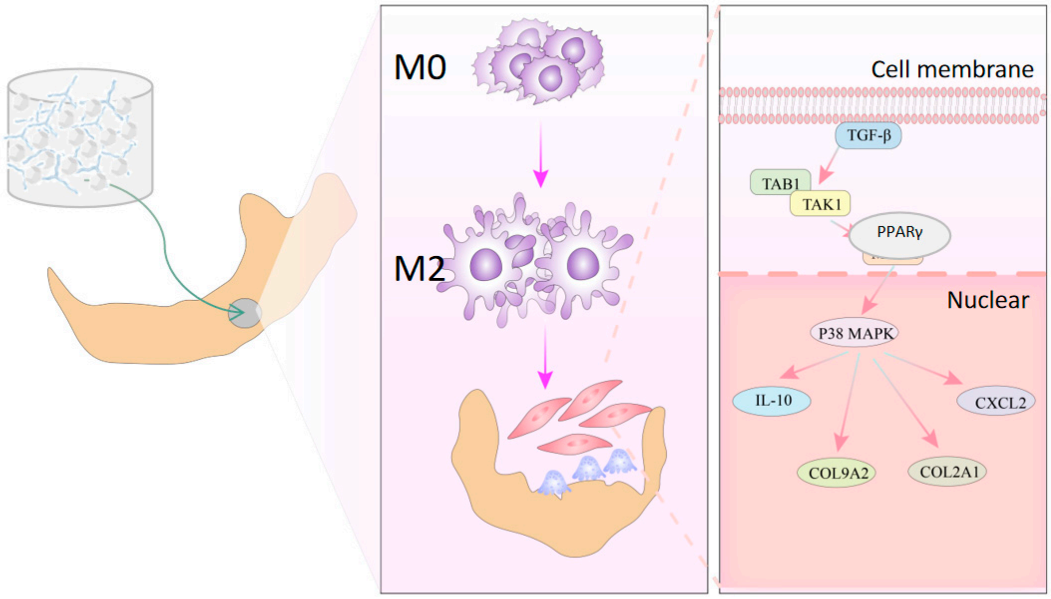

Figure S2. (A) Scheme of PCLLA-nanoHA material promotes M2 polarization of macrophages, partly through the enrichment of the TGF-β and PPARγ signaling pathways.
